# Zoonotic Potential of Non–*Helicobacter pylori* Helicobacter Species Associated With Dogs and Cats in Human Infections: A Systematic Review

**DOI:** 10.1155/vmi/3012354

**Published:** 2026-10-25

**Authors:** Nagham Mohammed Al-Gburi

**Affiliations:** ^1^ Zoonotic Diseases Research Unit College of Veterinary Medicine University of Baghdad Baghdad Iraq uobaghdad.edu.iq

**Keywords:** companion animals, *Helicobacter bizzozeronii*, *Helicobacter canis*, *Helicobacter felis*, *Helicobacter heilmannii*, *Helicobacter salomonis*, non–*Helicobacter pylori* helicobacter, One Health

## Abstract

**Background:**

Non–*Helicobacter pylori* Helicobacter (NHPH) spp. are clinically relevant organisms found in humans and several animal hosts. Dogs and cats are natural hosts of multiple gastric and enterohepatic NHPH spp. and may represent potential sources of human exposure.

**Objective:**

This systematic review aimed to synthesize the clinical, epidemiological, and molecular evidence concerning human infections involving dog‐ and cat‐associated NHPH spp. and to evaluate the evidence for a possible epidemiological association with companion animals.

**Methods:**

PubMed and Scopus databases were searched for studies published from 2000 through the final search on April 15, 2026. Eligible studies reported human infection with NHPH spp. involving *H. heilmannii* sensu stricto (s.s), *H. felis*, *H. bizzozeronii*, *H. salomonis*, or *H. canis*. The following information was extracted: study and patient characteristics, NHPH species, human samples, diagnostic techniques, molecular confirmation, immunological state, clinical results, and exposure to dogs and/or cats. The risk of bias was evaluated using the Joanna Briggs Institute (JBI) critical appraisal checklist appropriate for each study design.

**Results:**

Of 350 records identified, 51 studies met the inclusion criteria. The evidence consisted predominantly of case reports. *H. heilmannii*–related organisms were the most frequently reported, followed by *H. canis*, *H. felis*, *H. bizzozeronii*, and *H. salomonis*. Gastric NHPH infections were predominantly associated with gastric disease, while *H. canis* was mainly associated with systemic and extragastric infections, especially in immunocompromised or clinically vulnerable patients. Molecular confirmation was reported in 66.7% of studies (34/51), with one additional study providing molecular confirmation for only 2 of 22 cases. Dog and/or cat exposure was documented in 41.2% of studies (21/51). Although several studies identified the same NHPH spp. in humans and companion animals, paired strain‐level molecular evidence remained limited and was insufficient to establish the direction of transmission.

**Conclusion:**

Numerous gastrointestinal and systemic infections in humans have been linked to NHPH spp. found in dogs and cats. According to available data, dogs and cats may act as reservoirs or sources of human NHPH infection, given the recognized zoonotic potential of these pathogens. However, direct animal‐to‐human transmission and its direction have not been conclusively established. This is largely due to the limited availability of strain‐level molecular comparisons and paired human–animal sampling. To clarify transmission routes and improve integrated One Health surveillance, consistent molecular identification and comparative genomic studies are therefore required.

## 1. Introduction

*Helicobacter* spp. are Gram‐negative, spiral or curved, microaerophilic bacteria that colonize the gastrointestinal tract of humans and a wide variety of animal species. In addition to the human‐adapted *Helicobacter pylori*, several non–*H. pylori* Helicobacter (NHPH) spp. are increasingly recognized as clinically relevant in human and veterinary medicine. Historically, long spiral organisms observed in gastric tissues were collectively described as Helicobacter‐like organisms, *H. heilmannii*–like organisms, or *H. heilmannii* sensu lato (s.l.), because morphology alone did not permit reliable species‐level differentiation. Subsequent molecular investigations demonstrated that these organisms represent several distinct Helicobacter spp., leading to more precise species‐level classification and to the distinction between *H. heilmannii* sensu lato (s.l.) and *H. heilmannii* sensu stricto (s.s.) [1, 2]. NHPH spp. are broadly classified according to tissue tropism into gastric helicobacters, which colonize the stomach and include *H. felis*, *H. heilmannii* s.s., *H. salomonis*, and *H. bizzozeronii*, and enterohepatic helicobacters, which primarily inhabit the intestinal or hepatobiliary tract and include *H. bilis* and *H. canis* [3, 4].

Dogs and cats are important reservoirs of several gastric and enterohepatic NHPH spp. and may represent sources of human exposure. *H. heilmannii* s.s. and *H. felis* have been detected in both cats and dogs, whereas *H. bizzozeronii* and *H. salomonis* are predominantly associated with dogs. *H. canis* has also been detected in both dogs and cats. These animals may remain asymptomatically colonized, and transmission to humans may occur through direct or indirect contact with these animals; however, the specific transmission pathways and direction of transmission remain uncertain [1, 5, 6].

Human infections involving gastric NHPH spp. have been associated with chronic gastritis, dyspepsia, peptic ulcer disease, and gastric mucosa‐associated lymphoid tissue (MALT) lymphoma. In contrast, H. canis has primarily been associated with extragastric manifestations, such as bacteremia, cellulitis, hepatitis, and cholecystitis, particularly in immunocompromised patients [7–9].

The accurate identification of NHPH spp. remains challenging. Histopathological examination can detect long spiral organisms in gastric tissue but cannot reliably differentiate closely related NHPH spp. Culture is also difficult because many NHPH spp. are fastidious and require specialized growth conditions. Molecular methods, including species‐specific PCR, fluorescence in situ hybridization, and sequencing of targets such as the 16S rRNA, 23S rRNA, and ureAB genes, have improved species‐level identification [10–13]. More recent applications of next‐generation sequencing and whole‐genome sequencing have provided greater taxonomic resolution and revealed previously unrecognized diversity among gastric Helicobacter spp. from human and animal hosts [14, 15]. However, strain‐level genomic comparisons of epidemiologically linked humans and companion animals remain limited.

Previous reviews have broadly addressed the pathogenic and zoonotic significance of NHPH spp. in humans and animals [1, 5, 7, 9]. However, a systematic synthesis specifically focusing on documented human infections involving dog‐ and cat‐associated NHPH spp. remains limited, particularly with regard to species distribution, clinical manifestations, molecular identification, patient immune status, companion‐animal exposure, and evidence of possible zoonotic associations. Therefore, this systematic review aimed to synthesize the available clinical, epidemiological, and molecular evidence on human infections involving dog‐ and cat‐associated NHPH, specifically *H. heilmannii* s.s., *H. felis*, *H. bizzozeronii*, *H. salomonis*, and *H. canis*, and to evaluate the epidemiological evidence for a possible association with exposure to dogs and cats from a One Health perspective.

## 2. Materials and Methods

### 2.1. International Prospective Register of Systematic Reviews (PROSPERO) Registration and Reporting Guidelines

The review protocol was registered in the PROSPERO (CRD420261309381).

### 2.2. Information Sources and Search Strategy

A comprehensive literature search was conducted in PubMed and Scopus between February 15, 2026, and April 15, 2026, to identify eligible studies published from 2000 through the final search date of April 15, 2026. The same combination of keywords and Boolean operators was applied in both databases: (“Non‐Helicobacter pylori Helicobacter” OR NHPH OR “*Helicobacter felis*” OR “*Helicobacter heilmannii*” OR “*Helicobacter bizzozeronii*” OR “*Helicobacter salomonis*” OR “*Helicobacter canis*”) AND (human OR patient OR infection) AND (dog OR cat OR companion animal OR pet OR zoonotic). Only publications written in English were included. To identify potentially eligible studies not retrieved by the initial search, additional searches were conducted in PubMed and Scopus using broader combinations of relevant search terms. Eligible publication types included full‐text articles, case reports, case series, observational studies, and published abstracts that provided sufficient information for eligibility assessment and data extraction. The literature search was limited to PubMed and Scopus databases because access to other subscription‐based databases, including Web of Science and Embase, was unavailable.

### 2.3. Study Design and Selection

This systematic review was conducted and reported in accordance with the Preferred Reporting Items for Systematic Reviews and Meta‐Analyses (PRISMA) 2020 guidelines [16]. All records retrieved from PubMed and Scopus were imported into Rayyan, a web‐based platform for systematic review screening [17]. Rayyan was used to identify and remove duplicate records and to manage title and abstract screening and full‐text eligibility assessment. One reviewer screened all records, assessed the available informative reports according to the eligibility criteria, and performed data extraction and methodological quality assessment. The study selection process was documented using a PRISMA 2020 flow diagram. ChatGPT (OpenAI) was used as an assistive tool for structuring the literature review and extracting study data; the extracted data were then reviewed and verified by the author.

### 2.4. Inclusion and Exclusion Criteria

#### 2.4.1. Inclusion Criteria

• Human studies involving *H. felis*, *H. heilmannii* s.s, *H. salomonis*, *H. bizzozeronii*, or *H. canis.*
• Identification of the NHPH spp. using an appropriate diagnostic method.
• Information on exposure or nonexposure to dogs and/or cats, when available.
• Sufficient clinical, epidemiological, or microbiological information to confirm eligibility and permit extraction of the relevant study data.
• Full‐text articles and published abstracts with sufficient information for inclusion.

#### 2.4.2. Exclusion Criteria

• Studies focusing exclusively on *H. pylori* infection.
• Animal‐only studies with no human data.
• Studies in which the target NHPH spp. were detected exclusively in animals, with no corresponding human infection.
• Non‐English publications.
• Non–peer‐reviewed publications.
• Reviews without original human cases or data.
• Abstracts with insufficient information to confirm eligibility or permit the extraction of the relevant study data.

### 2.5. Data Extraction and Classification

• Study reference and country.
• Study design.
• Number of human cases or study participants.
• Identified NHPH species and reported detection rates, when available.
• Type of human sample, including gastric biopsy or tissue, blood, stool, and saliva.
• Diagnostic methods.
• Molecular confirmation status.
• Patient characteristics.
• Immune status.
• Clinical manifestations and outcomes.
• Documented exposure or nonexposure to dogs and/or cats.

### 2.6. Risk‐of‐Bias Assessment

The quality of the included studies was evaluated by the reviewer using the JBI critical appraisal checklist appropriate to each study design [18]. Each appraisal item was rated as “Yes,” “No,” “Unclear,” or “Not applicable.” An “Unclear” rating was assigned when the available information was insufficient to make a judgment, particularly for publications available only as abstracts.

### 2.7. Data Synthesis and Statistical Analysis

The findings were synthesized narratively and presented in structured tables. Comparable data were summarized using frequencies and percentages. No inferential statistical analysis was performed. Meta‐analysis was not conducted because of heterogeneity in study designs, participant characteristics, diagnostic methods, and reported outcomes.

## 3. Results

A total of 350 records were identified, of which 51 studies were included in this systematic review (Figure 1; Table 1). The included studies were published between 2000 and 2026. The included studies represented five geographical regions, with the largest proportion originating from Europe (24/51, 47.1%), followed by Asia (15/51, 29.4%), North America (9/51, 17.6%), Africa (2/51, 3.9%), and Oceania (1/51, 2.0%).

**FIGURE 1:**
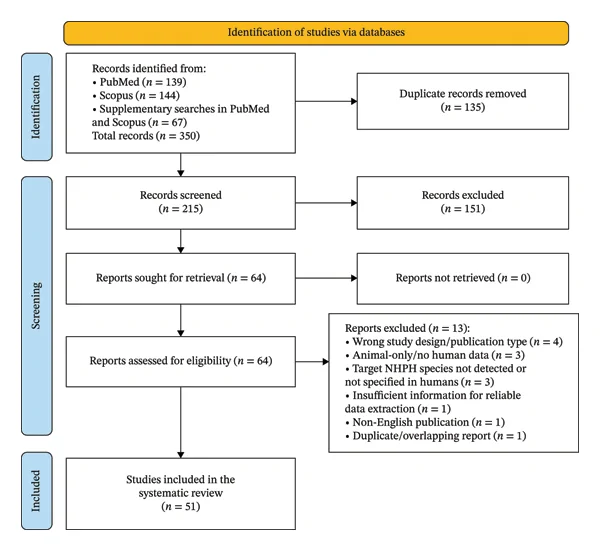
PRISMA 2020 flow diagram showing the identification, screening, eligibility assessment, and inclusion of studies in the systematic review.

**TABLE 1:** Characteristics of the studies included in the systematic review.

Study	Country	Study type/human population	Species detected	Human sample	Diagnostic method	Molecular confirmation	Patient/population characteristics	Immune status	Clinical outcome/findings	Animal exposure
[13]	Belgium	Retrospective/prospective; 464 retrospective, 65 prospective, 38 controls	*H. bizzozeronii; H. felis*; *H. salomonis*; *H. heilmannii* s.s.	Gastric biopsies; cytobrush; saliva	Histopathology; genus/species PCR	Yes	Adults with gastric NHPH infection and controls	NR	Chronic gastritis; PUD; MALT lymphoma	Exposure assessed; no significant association
[19]	Japan	Case report	*H. heilmannii*	Gastric biopsy	Endoscopy; histology; Giemsa; qPCR	Yes	Woman in her 40s	Previous cervical cancer; immunodeficiency NR	Cobblestone gastritis; synchronous early gastric cancers	Long‐term dog/cat ownership
[20]	Japan	Case report	*H. heilmannii* s.s.	Gastric biopsy	Histology; IHC; PCR	Yes	69‐year‐old man	NR	Gastric MALT lymphoma	Historical dog exposure (> 20 years); no dog/cat ownership in previous 5 years
[21]	Slovenia	Case report	*H. canis*	Blood	Culture; MALDI‐TOF; 16S rRNA sequencing	Yes	65‐year‐old woman	RA; immunosuppression; hypogammaglobulinemia	Bacteremia; recovered	Close contact with pet dogs
[10]	Germany	Molecular series; 89 positive biopsies	NHPH including *H. salomonis*‐related organisms	Gastric biopsies	Histology; FISH; PCR; partial 16S rRNA sequencing	Yes	HHLO‐positive patients	NR	Gastritis; NHPH colonization	NR
[22]	Portugal	Cross‐sectional; *n* = 80	*H. felis* (2/80)	Gastric tissue	Giemsa; IHC; PCR; sequencing	Yes	Two *H. felis*–positive patients; both obese	NR	Mixed *H. pylori/H. felis* infection	Cat contact in one positive patient
[23]	Netherlands	Case within three‐case series	*H. canis*	Blood	Culture; MALDI‐TOF; 16S rRNA sequencing	Yes	49‐year‐old woman	RA; immunosuppressive therapy	Cellulitis; bacteremia; recovered	Dog owner; no recalled bite/scratch
[24]	Japan	Case report	*H. heilmannii* s.s.	Gastric biopsies	Species‐specific PCR	Yes	44‐year‐old woman	Mixed connective tissue disease; long‐term prednisolone	Enlarged gastric hyperplastic polyps; one with malignant transformation	Historical dog exposure (> 20 years); none since age 22
[25]	Australia	Case report	*H. canis*/Helicobacter‐like organism	Blood	Culture; 16S rRNA sequencing	Yes	27‐year‐old man	X‐linked hypogammaglobulinemia	Recurrent bacteremia; fever; multifocal cellulitis	Possible exposure to young dog
[26]	Mexico	Case report	*H. bizzozeronii*	Gastric biopsy	H&E; Warthin–Starry; ureA PCR; sequencing	Yes	20‐month‐old girl	No previous illness	Hemorrhagic gastropathy; gastric ulcer; chronic‐active gastritis	Close, frequent contact with family dog
[27]	USA	Case report	*H. heilmannii*	NR	NR in abstract	No	Adult man	Well‐controlled HIV	Gastritis; successful eradication	NR
[28]	Iran	Cross‐sectional; *n* = 80	*H. heilmannii*	Gastric biopsies	RUT; PCR	Yes	Patients undergoing gastric evaluation	NR	Gastric infection; clinical associations assessed	NR
[29]	Netherlands	Case report	*H. canis*	Blood	Blood culture; 16S rRNA sequencing	Yes	41‐year‐old woman	Kidney transplant recipient	High fever; bacteremia; recovered	NR
[30]	Iran	Cross‐sectional; 428 screened; *n* = 60 analyzed	*H. felis; H. salomonis*; *H. heilmannii*; *H. bizzozeronii*	Gastric biopsies	RUT; H&E; Giemsa; PCR; qPCR	Yes	Adults with gastric disease	NR	Gastritis; gastric/duodenal ulcer	Exposure variably reported
[31]	Japan	Case report	*H. heilmannii*	Gastric biopsy	Histopathology; PCR	Yes	46‐year‐old man	NR	Gastric MALT lymphoma; remission after eradication	NR
[32]	Serbia	Case report	*H. heilmannii*	Gastric biopsy	Endoscopy; histopathology; modified Giemsa	No	50‐year‐old woman	No immunocompromising condition reported	Dyspepsia; gastritis; improved after therapy	NR
[33]	USA	Case report	*H. canis*	Blood	Culture; MALDI‐TOF; 16S rRNA sequencing	Yes	57‐year‐old woman	CVID; prednisone; prior splenectomy	Recurrent fever; bacteremia; recovered	Two dogs; three cats
[34]	Germany	Case report	*H. felis*	Gastric biopsy	Culture; phylogenetic analysis	Yes	14‐year‐old girl	NR	Persistent epigastric pain; gastric *H. felis* isolation	NR
[35]	USA	Case series; *n* = 2	*H. heilmannii*	Antral biopsies	Endoscopy; histopathological morphology	No	11‐year‐old boy; 2‐year‐old girl	NR	Gastritis; epigastric pain; failure to thrive	NR
[36]	Denmark	Case report	*H. canis*	Blood	Culture; phenotypic ID; 16S rRNA sequencing	Yes	7‐month‐old girl	Otherwise healthy	Intermittent fever; bacteremia; treated	Household cat; no dog
[37]	France	Case report	*H. heilmannii*	Gastric biopsy	Histopathology; morphology	No	Adult; age/sex NR	Polymyositis; immunosuppressive context	Severe gastritis; symptoms resolved	NR
[38]	USA	Case report	*H. heilmannii*	Antral biopsy	NR in abstract	No	70‐year‐old woman	NR	Gastric‐antral colonization; gastritis	NR
[39]	Italy	Case report	*H. heilmannii*	Antral/duodenal‐bulb biopsies	Histological/morphological ID	No	Adult; age/sex NR	NR	Infection of antrum and duodenal bulb	NR
[40]	USA	Case report	*H. heilmannii*	Antral biopsy	H&E; anti‐*H. pylori* IHC	No	44‐year‐old man	NR	Mild chronic gastritis; lymphoid aggregates	NR
[41]	Canada	Case report	*H. heilmannii*	NR	NR in abstract	No	50‐year‐old man	NR	Acute febrile gastritis; successfully treated	NR
[42]	Poland	Pediatric case series; *n* = 12	*H. heilmannii*; 3 *H. pylori* coinfections	Gastric/gastroduodenal biopsies	Endoscopy; microscopy; culture; histopathology	No	7 boys, 5 girls; 9–17 years	NR	Gastritis; duodenitis; one duodenal ulcer; symptoms resolved	NR
[43]	South Africa	Cross‐sectional; *n* = 90	*H. felis*	Gastric biopsies	Histopathology; ureA/ureB PCR; sequencing	Yes	33 children; 57 adults	NR	*H. felis* detection; frequent *H. pylori* coinfection	NR
[44]	Czech Republic	Prevalence study; *n* = 33	*H. heilmannii*	Gastric biopsies	Histology; morphology	No	Predominantly rural population	NR	2% prevalence; chronic‐active gastritis	Dog/cat contact NR; rural exposure reported
[45]	Portugal	Cross‐sectional; *n* = 54	*H. bizzozeronii* (14/54)	Antral/corpus biopsies	Histology; Giemsa; species‐specific PCR; Sanger sequencing	Yes	Adults ≥ 18 years	NR	Molecularly confirmed infection; histological gastritis	NR
[46]	Japan	Prevalence study; 4074 EGD; 19 clinically analyzed	*H. heilmannii*	Gastric biopsies	H&E; modified McMullen; IHC; TEM in subset	No	Adults undergoing EGD	NR	Chronic gastritis; MALT lymphoma	NR
[47]	South Korea	Comparative cross‐sectional; *n* = 10	*H. heilmannii* morphology	Gastric biopsies	H&E; Wright–Giemsa; IHC; morphology	No	10 patients; mean age 49 years	NR	Chronic gastritis; erosions/ulcer; one MALT lymphoma	Dog 4/10; pig 1/10; none 5/10
[48]	Japan	Case report	*H. heilmannii*	Antral mucosa	Morphology; urease; culture negative	No	11‐year‐old boy	NR	Infection 3 years after *H. pylori* ulcer; eradicated	No dog/cat contact
[11]	Belgium/Germany	Molecular study; 126 patients; 123 evaluable	*H. salomonis*; *H. felis*; *H. heilmannii* s.s.; *H. bizzozeronii*	FFPE gastric biopsies	Histology; group PCR; multiplex species‐specific PCR	Yes	Histologically identified NHPH cases	NR	Species characterization of gastric NHPH	Human, dog, cat populations studied; no individual ownership linkage
[49]	Bulgaria	Pediatric observational/prevalence; *n* = 658	*H. heilmannii* (2/658)	Antral specimens	Endoscopy; microbiological/morphological ID	No	658 untreated children	NR	Two pediatric infections	NR
[50]	Switzerland	Case report	*H. canis*	Blood	Culture; biochemical ID; 16S rRNA sequencing	Yes	44‐year‐old man	Immunocompetent	Bacteremia; multifocal cellulitis; recovered	Two pet cats; occasional dog contact
[12]	Belgium/Germany	Molecular series; 108 HHLO‐positive; 101 evaluable	*H. bizzozeronii*; *H. salomonis*; *H. felis*; other NHPH	FFPE gastric biopsies	Histology; species‐specific 16S PCR; multiplex PCR	Yes	42 women; 66 men	NR	Canine/feline‐associated spp. in 49/101; mixed infection 11/101	NR
[51]	Belgium	Paired human–dog case report	*H. felis*	Gastric biopsy	Histology; multiplex PCR	Yes	Elderly woman	NR	Severe gastric ulceration	Pet dog; *H. felis* also detected in dog
[52]	France	Case report	*H. canis*	Duodenal biopsy	Culture; *Helicobacter*‐specific PCR; 16S rRNA sequencing	Yes	52‐year‐old woman	Crohn’s disease; diabetes	Chronic duodenal ulcerations	Historical cat exposure
[53]	Finland	Case report; multiple isolates from one patient	*H. bizzozeronii*	Gastric biopsies	Culture; microscopy; partial 16S; ureAB; sAFLP	Yes	Adult; age/sex NR	NR	Severe dyspepsia; chronic‐active gastritis; persistent resistant clone	NR
[54]	Japan	Case report	*H. heilmannii* s.s.	Gastric biopsy	Histology; IHC; TEM; 16S/urease sequencing	Yes	62‐year‐old woman	No immune disorder reported	Multiple gastric ulcers; remission after eradication	Three household cats
[55]	USA	Case report	*H. canis*	Blood	Culture; 16S rRNA sequencing; gyrA analysis	Yes	49‐year‐old man	ESRD; hemodialysis	Bacteremia; cellulitis near AV fistula	Close contact with pet dog
[56]	China	Prevalence study; *n* = 1499	*H. felis; H. bizzozeronii*; *H. heilmannii* s.s.; *H. salomonis*	Gastric biopsies	Species‐specific PCR; sequencing	Yes	*H. pylori*‐positive patients with gastric disease	NR	NHPH coinfection 178/1499	NR
[57]	Belgium	Case report	*H. felis*	Gastric biopsies	H&E; IHC; species‐specific qPCR	Yes	39‐year‐old man	No relevant immune disorder	Severe acute gastritis; focal necrosis; treated	No dog/cat contact; guinea pig/parakeet reported
[58]	Iran	Analytical cross‐sectional; *n* = 190	*H. heilmannii*; *H. bizzozeronii*	Antral biopsies	Endoscopy; species‐specific PCR	Yes	Mean age 52 years	NR	Gastritis; duodenal ulcer; one gastric cancer	NR
[59]	South Korea	Analytical cross‐sectional human–dog study; 124 humans, 39 dogs	*H. felis*; *H. bizzozeronii*	Saliva; feces	Genus/species nested PCR; sequencing	Yes	Dog owners, veterinarians, non‐owners	NR	Mainly subclinical	Dog exposure; 39 owner–dog pairs; species‐level comparison, no strain‐level genomic matching
[60]	USA	Retrospective pediatric case series; *n* = 38	*H. heilmannii*	Gastric biopsies	Histopathology; morphology	No	38 children; 1–18 years	NR	Pain; nausea/vomiting; gastric nodularity; chronic gastritis	NR
[61]	Poland	Prevalence study; 22 infected children	*H. heilmannii*	Antral biopsies	EGD; microscopy/Gram stain; culture; PCR in 2/22	Partial (2/22)	11 girls, 11 boys; 4–18 years	NR	Pain 22/22; nodular gastritis 17/22; duodenitis 5/22; eradication 11/11 followed	Dog/cat contact 12/22 (54.5%)
[62]	India	Case report	*H. heilmannii*	Gastric‐body biopsy	Endoscopy; H&E; Giemsa; Warthin–Starry; IHC	No	70‐year‐old man	NR	Chronic gastritis; atrophy; metaplasia; lymphoid follicles	Domestic‐animal exposure; species unspecified
[63]	Pakistan	Analytical cross‐sectional; *n* = 250	*H. heilmannii* 17/250; *H. felis* 10/250	Antral/corpus biopsies	EGD; RUT; histology; PCR; sequencing	Yes	Mean age 39 ± 12 years	NR	Gastritis; duodenal ulcer; intestinal metaplasia in some patients	Dog/cat exposure 66/250 overall; 1/17 *H. heilmannii*‐ and 2/10 *H. felis*–positive exposed
[64]	Egypt	Comparative cross‐sectional human–animal study; 70 humans, 70 dogs, 65 cats	Human *H. heilmannii*: 1/30 stool, 1/10 fundus biopsies; *H. felis* absent	Serum; stool; fundus biopsies	Culture; Gram stain; biochemistry; serology; PCR	Yes	Patients with GI disorders	NR	Two positive human samples; diagnoses not individually linked	Contact with studied dogs/cats; individual pairing not established
[8]	Belgium	Case report	*H. canis*	Blood	Blood culture/subculture; partial 16S rRNA sequencing	Yes	55‐year‐old woman	Kidney transplant; immunosuppression; previous splenectomy	Bacteremia; clinical/bacteriological cure after 6‐week IV ceftriaxone	Close contact with pet dog; dog stool culture negative

*Note:* AV, arteriovenous; CVID, common variable immunodeficiency; EGD, esophagogastroduodenoscopy; GI, gastrointestinal; ID, identification; IHC, immunohistochemistry; IV, intravenous; MALDI‐TOF, matrix‐assisted laser desorption/ionization time‐of‐flight mass spectrometry; sAFLP, single‐enzyme amplified fragment length polymorphism.

Abbreviations: ESRD, end‐stage renal disease; FFPE, formalin‐fixed paraffin‐embedded; FISH, fluorescence in situ hybridization; H&E, hematoxylin and eosin; HHLO, *Helicobacter heilmannii*–like organism; MALT, mucosa‐associated lymphoid tissue; NHPH, non–*Helicobacter pylori Helicobacter*; NR, not reported; PCR, polymerase chain reaction; PUD, peptic ulcer disease; qPCR, quantitative PCR; RA, rheumatoid arthritis; rRNA, ribosomal RNA; RUT, rapid urease test; TEM, transmission electron microscopy.

The included studies comprised case reports, case series, prevalence studies, cross‐sectional studies, and molecular investigations. Both adult and pediatric populations were represented. The studies reported human infections involving the gastric NHPH spp. *H. heilmannii* s.s., *H. felis*, *H. bizzozeronii*, and *H. salomonis*, as well as the enterohepatic sp. *H. canis*. The characteristics of the included studies, including study design, human population, NHPH spp., sample type, diagnostic method, molecular confirmation, patient characteristics, immune status, clinical findings or outcomes, and animal exposure are summarized in Table 1.

Most of the included studies focused on gastric NHPHs, whereas *H. canis* was reported mainly in case reports of extragastric infections. Among the gastric NHPHs, *H. heilmannii* and *H. heilmannii*–like organisms were the most frequently reported, followed by *H. felis*, *H. bizzozeronii*, and *H. salomonis*. However, several studies relied primarily on bacterial morphology and reported *H. heilmannii*–like organisms without definitive species‐level identification. Molecular studies identified single or mixed infections involving two or more Helicobacter spp. [11–13, 30, 56]. Gastric biopsy specimens obtained during upper gastrointestinal endoscopy were the primary samples used to detect gastric NHPHs. Histopathology, immunohistochemistry, PCR, and nucleotide sequencing were used either individually or in combination. Molecular confirmation using PCR and/or nucleotide sequencing was reported in 34 of the 51 included studies (66.7%). In one additional study, PCR was performed in only 2 of 22 cases and was therefore classified as partial molecular confirmation. Blood cultures followed by molecular identification were used mainly in reports of systemic *H. canis* infection. Other samples evaluated in a smaller number of studies included saliva, buccal swabs, and stool specimens.

Clinical manifestations associated with gastric NHPH infections (*H. heilmannii*, *H. felis*, and *H. bizzozeronii*) included chronic or chronic‐active gastritis, dyspepsia, abdominal discomfort, nausea, vomiting, peptic or gastric ulcers, and gastric MALT lymphoma [26, 30, 31, 46, 47, 53, 60, 61, 63]. *H. canis* was mainly associated with extragastric and systemic infections, including bacteremia, recurrent fever, and cellulitis. Many of these infections were reported in immunocompromised or clinically vulnerable patients [21, 25, 29, 33, 36, 50, 55]. Similar clinical manifestations were also reported in pediatric patients [36, 48, 49, 60, 61].

In histopathological examinations, gastric NHPH infections were mainly characterized by chronic or chronic‐active gastritis with mononuclear inflammatory cell infiltration and varying degrees of neutrophilic activity [26, 46, 47, 53, 61, 63]. The organisms were frequently detected in the gastric mucus layer, within the foveolae, or in glandular lumina [26, 46, 47, 57]. In children, histological evidence of gastritis was also observed despite normal or nonspecific endoscopic findings [60, 61]. Yakoob et al. [63] reported intestinal metaplasia in 25% (2/8) of patients coinfected with *H. pylori* and *H. heilmannii* and in 20% (1/5) of those coinfected with *H. pylori* and *H. felis*, compared with 3% (3/94) of patients infected with *H. pylori* alone. Gastric MALT lymphoma was also reported in association with *H. heilmannii* infection [31, 46, 47]. Kamio et al. [19] reported *H. heilmannii*–associated gastritis characterized by predominant lymphocytic infiltration and relatively low neutrophilic activity in a patient with multiple synchronous early gastric cancers.

Among the 51 included studies, 21 (41.2%) documented current, previous, or possible exposure to dogs and/or cats. These studies included case reports, pediatric case series, and observational studies [8, 19–26, 33, 36, 47, 50–52, 54, 55, 59, 61, 63, 64]. However, information on companion‐animal exposure was unavailable in many of the included studies. Several studies also investigated NHPH spp. in both humans and companion animals [11, 51, 59, 64]. Van den Bulck et al. [11] detected several gastric NHPH spp. in human, dog, and cat populations. De Bock et al. [51] detected *H. felis* in both a patient and her pet dog. Chung et al. [59] detected *H. felis* and *H. bizzozeronii* in humans and dogs and reported an association between dog contact and human *H. felis* infection. Moussa et al. [64] detected *H. heilmannii* in humans, dogs, and cats, whereas *H. felis* was not detected.

### 3.1. Risk‐of‐Bias Assessment

The risk of bias of all 51 included studies was evaluated using the JBI critical appraisal checklist appropriate to each study design, including 29 case reports, six case series, eight analytical cross‐sectional studies, seven prevalence studies, and one mixed observational/cohort study. Overall, full‐text case reports provided adequate clinical and diagnostic information, although reporting of adverse events, follow‐up, immune status, and animal exposure was inconsistent. The main limitations of case series were unclear case inclusion and recruitment methods, while cross‐sectional studies had insufficient control of confounding factors and, at times, unclear sampling methods. Prevalence studies were mainly limited by sampling frames, population representativeness, and insufficient justification of sample size. Species confirmation also varied, ranging from histopathological morphology to species‐specific PCR or sequencing. Nineteen publications were available only as sufficiently informative abstracts; therefore, items that could not be confirmed were rated as “Unclear” rather than “No.” No study was excluded solely based on the appraisal results (Tables S1–S5).

## 4. Discussion

Dogs and cats are natural hosts and potential reservoirs of several gastric NHPH spp., including *H. heilmannii* s.s., *H. felis*, *H. bizzozeronii*, and *H. salomonis*. These organisms commonly colonize the gastric mucosa of companion animals, frequently without evident clinical signs. The high colonization rates reported in dogs and cats, together with the detection of these animal‐associated NHPH spp. in human gastric specimens, support a possible zoonotic association [1, 5, 11, 12].

Gastric NHPH spp. predominated among the organisms reported in the included studies, particularly *H. heilmannii* and *H. heilmannii*–like organisms, followed by *H. felis*, *H. bizzozeronii*, and *H. salomonis*. Historically, long, tightly coiled gastric *Helicobacter* organisms were often classified collectively as *H. heilmannii* or *H. heilmannii*–like organisms based on histopathological morphology. However, several gastric NHPH spp. are morphologically similar and cannot be reliably differentiated by routine microscopy. The subsequent use of species‐specific PCR, sequencing, and fluorescence in situ hybridization enabled more accurate species‐level identification [10–13]. Therefore, although *H. heilmannii–*related organisms were the most frequently reported in this review, historical diagnostic grouping may have influenced the reported distribution of individual gastric NHPH spp.

The clinical patterns identified in this review differed according to NHPH spp. Gastric NHPH spp. were predominantly associated with inflammatory and ulcerative gastric diseases, including chronic or chronic‐active gastritis, dyspepsia, peptic or gastric ulceration, and, less frequently, gastric MALT lymphoma. This clinical pattern is consistent with the gastric colonization of these organisms. The clinical spectrum of gastric NHPH infection partially overlaps with that of *H. pylori*, emphasizing the importance of considering NHPH in patients with unexplained gastric disease, particularly when *H. pylori* is not detected [13, 46, 47, 61]. The reported association with gastric MALT lymphoma is clinically important, although the limited number of cases and variability in species identification preclude conclusions regarding species‐specific risk [31, 46, 47]. Similarly, occasional reports of gastric cancer in NHPH‐positive patients warrant further investigation but do not establish a causal relationship [19].

In contrast, *H. canis* showed a predominantly extragastric and systemic clinical profile, with much of the available evidence derived from individual case reports. Reported manifestations included bacteremia, recurrent fever, and cellulitis, with other invasive infections also described. This difference is consistent with the distinct biological characteristics and tissue tropism of *H. canis* as an enterohepatic rather than a gastric *Helicobacter* species [3, 8]. Many invasive *H. canis* infections occurred in immunocompromised or clinically vulnerable patients, including individuals with hypogammaglobulinemia, malignancy, transplantation, or immunosuppressive therapy [21, 25, 29, 33, 55]. This pattern suggests that impaired host defenses may facilitate bloodstream invasion and systemic disease. However, *H. canis* infection has also been reported in patients without recognized severe immunosuppression, indicating that immune status alone does not explain susceptibility [50].

Dogs and cats commonly harbor gastric NHPH spp. and may remain asymptomatically colonized. Some of these organisms have also been detected in the oral cavity and saliva of companion animals. Close contact with colonized companion animals, including exposure to saliva or vomitus, represents a biologically plausible route of NHPH transmission. Exposure to fecal material and indirect environmental contamination has also been proposed; however, the relative importance of these potential routes remains uncertain [1, 22].

Documented dog and/or cat exposure in a substantial proportion of the included literature, together with reports of the same *Helicobacter* spp. in humans and companion animals, provides epidemiological support for a possible zoonotic association. In particular, the associations reported between continuous dog contact and human *H. felis* and *H. bizzozeronii* infections strengthen the epidemiological plausibility of companion‐animal exposure [59]. Nevertheless, species‐level concordance between humans and animals does not equate to evidence of transmission. Establishing zoonotic transmission requires stronger epidemiological linkage and strain‐level molecular or genomic comparison.

Furthermore, dog‐ and cat‐associated NHPH spp. have been identified in patients without documented companion‐animal contact [10, 12, 48], suggesting that unrecognized previous contact, other animal reservoirs, or indirect environmental exposure may also contribute. Thus, the current evidence supports the zoonotic potential of dog‐ and cat‐associated NHPH but does not establish direct or exclusive animal‐to‐human transmission.

The geographical distribution of the available evidence also warrants careful interpretation. The predominance of studies from Europe (47.1%) and Asia (29.4%), compared with the limited evidence from North America, Africa, and Oceania, may reflect differences in clinical awareness, access to endoscopy and molecular diagnostic methods, research capacity, and publication practices rather than true geographical differences in NHPH occurrence. Consequently, the available evidence does not permit reliable comparisons of regional incidence or prevalence. This geographical imbalance highlights the need for further studies in underrepresented regions using standardized diagnostic and epidemiological approaches.

From a One Health perspective, the detection of related NHPH spp. in both humans and companion animals highlights the importance of integrating human, veterinary, and molecular investigations. Future studies should include standardized species‐level identification, systematic assessment of animal exposure, paired sampling of patients and their companion animals, and detailed genomic comparisons. Such approaches would help distinguish simple host sharing of the same NHPH spp. from epidemiologically linked infections, providing stronger evidence of strain relatedness and possible transmission pathways. Integrated human–animal surveillance, together with evidence‐based education of pet owners regarding routine hygiene during close contact with companion animals, may also be considered within a One Health framework, although specific preventive measures require stronger evidence regarding transmission routes. The overall evidence should also be interpreted in light of its methodological limitations. The predominance of case reports, case series, and relatively small observational studies provides valuable information on uncommon clinical presentations and generates hypotheses regarding zoonotic exposure, but offers limited evidence for population‐level risk or causal transmission relationships. Similarly, insufficient consideration of potential confounding factors in several analytical studies limits the interpretation of associations between companion‐animal exposure and human infection. Consequently, the available evidence supports zoonotic potential and suggests a possible epidemiological association, while stronger prospective and molecular epidemiological studies are required before direct animal‐to‐human transmission can be established.

## 5. Limitations

This review has several limitations that should be considered when interpreting its findings. Most of the available evidence was derived from case reports, case series, and small observational studies. Consequently, the current evidence provides limited support for causal inference, assessment of risk factors, or reliable estimation of population prevalence. Considerable heterogeneity was also observed across studies in terms of study design, patient characteristics, diagnostic approaches, and reported outcomes, precluding quantitative pooling and meta‐analysis. Nineteen publications were available only as sufficiently informative abstracts and contained adequate information for eligibility assessment and data extraction. Nevertheless, the absence of full‐text reports prevented complete verification of methodological details and resulted in a greater number of “Unclear” judgments during critical appraisal. Diagnostic approaches were also inconsistent across studies. In particular, some reports identified gastric NHPH based on histopathological morphology without molecular confirmation at the species level, which may have affected the accurate differentiation of morphologically similar gastric NHPH spp. Information regarding exposure to dogs and cats was not consistently reported, while paired sampling of patients and their companion animals was uncommon. Furthermore, strain‐level genomic comparisons were rarely performed. Therefore, the detection of the same NHPH spp. in humans and companion animals could not distinguish direct transmission from exposure to a shared source or independent acquisition. The direction of any potential transmission also remains unresolved. Study selection, data extraction, and methodological quality assessment were undertaken by a single reviewer, introducing the possibility of selection bias and subjective judgment. In addition, the search was restricted to PubMed and Scopus and to peer‐reviewed publications in English. Relevant evidence indexed exclusively in other databases or published in other languages may therefore have been missed. Publication bias should also be considered because unusual or clinically important NHPH infections may be more likely to appear in the published literature than uncomplicated or asymptomatic infections. Finally, the geographical concentration of studies in Europe and Asia limits the generalizability of the findings to underrepresented regions.

## 6. Conclusion

This systematic review synthesized the available evidence on human infections involving dog‐ and cat‐associated NHPH spp., particularly *H. heilmannii* s.s., *H. felis*, *H. bizzozeronii*, *H. salomonis*, and *H. canis*. These organisms have been reported across a spectrum of gastric and systemic infections in humans. The available evidence supports their zoonotic potential and suggests a possible epidemiological association with companion‐animal exposure; however, direct animal‐to‐human transmission has not been conclusively established. Further studies incorporating systematic exposure assessment, paired sampling of humans and their companion animals, and strain‐level genomic comparisons are needed to clarify strain relatedness and potential transmission pathways. Integrating these approaches within a One Health framework may improve understanding of the epidemiology and public health significance of companion‐animal‐associated NHPH infections.

## Funding

No funding was received for this research.

## Disclosure

The author has read and approved the final version of the manuscript. The author had full access to all of the data in this study and takes complete responsibility for the integrity of the data and the accuracy of the data analysis. The author affirms that this manuscript is an honest, accurate, and transparent account of the study being reported; that no important aspects of the study have been omitted; and that any discrepancies from the study as planned and registered have been explained.

## Conflicts of Interest

The authors declare no conflicts of interest.

## Supporting Information

Additional supporting information can be found online in the Supporting Information section.

## Supporting information


**Supporting Information** Supporting file S1. Risk‐of‐bias assessment of the studies included in the systematic review. The file contains five supporting tables.

## Data Availability

All data generated or analyzed during this study are included in this published article and its Supporting Information files.
