# Supplementary material for: Zoonotic Potential of Non–Helicobacter pylori Helicobacter Species Associated With Dogs and Cats in Human Infections: A Systematic Review
Source: Vet Med Int. 2026 Oct 25;2026:3012354. doi: 10.1155/vmi/3012354 (PMC13695837; doi:10.1155/vmi/3012354)
Supplement: Supplementary file 1 [file VMI-2026-3012354-s001.docx]

Supplementary file S1

51 included studies – Joanna Briggs Institute (JBI) design-specific critical appraisal

**Table S1. JBI Critical Appraisal of Case Reports**

| **Study** | **Q1** | **Q2** | **Q3** | **Q4** | **Q5** | **Q6** | **Q7** | **Q8** | **Appraisal** |
| --- | --- | --- | --- | --- | --- | --- | --- | --- | --- |
| Kamio et al., 2025 | Y | Y | Y | Y | Y | Y | U | Y | Include |
| Fujihara et al., 2026 | Y | Y | Y | Y | Y | Y | U | Y | Include |
| Mihevc et al., 2021 | Y | Y | Y | Y | Y | Y | U | Y | Include |
| van Wezel et al., 2022 | Y | Y | Y | Y | Y | Y | U | Y | Include |
| Kurimoto et al., 2025 | Y | Y | Y | Y | Y | Y | U | Y | Include |
| Gerrard et al., 2001* | Y | U | Y | U | Y | U | U | Y | Include |
| Montijo-Barrios et al., 2023 | Y | Y | Y | Y | Y | Y | U | Y | Include |
| Granger & Knights, 2023* | U | U | Y | U | Y | Y | U | Y | Include |
| van der Vusse et al., 2014* | Y | Y | Y | Y | Y | Y | U | Y | Include |
| Okamura et al., 2013* | Y | Y | Y | Y | Y | Y | U | Y | Include |
| Vukojević et al., 2011 | Y | Y | Y | Y | Y | Y | U | Y | Include |
| Abidi et al., 2013 | Y | Y | Y | Y | Y | Y | U | Y | Include |
| Wüppenhorst et al., 2013* | Y | U | Y | Y | U | U | U | Y | Include |
| Prag et al., 2007 | Y | Y | Y | Y | Y | Y | U | Y | Include |
| Marie et al., 2006* | U | U | Y | Y | Y | Y | U | Y | Include |
| Hazra et al., 2006* | Y | U | U | U | U | U | U | Y | Include |
| Monno et al., 2006* | U | U | Y | Y | U | U | U | Y | Include |
| Singhal & Sepulveda, 2005* | Y | Y | Y | Y | U | U | U | Y | Include |
| Al-Hamoudi et al., 2006* | Y | Y | Y | U | Y | Y | U | Y | Include |
| Kato et al., 2005* | Y | Y | Y | Y | Y | Y | U | Y | Include |
| Leemann et al., 2006 | Y | Y | Y | Y | Y | Y | U | Y | Include |
| De Bock et al., 2007* | Y | Y | Y | Y | U | U | U | Y | Include |
| Tankovic et al., 2011 | Y | Y | Y | Y | Y | Y | U | Y | Include |
| Kivistö et al., 2010* | U | Y | Y | Y | Y | Y | Y | Y | Include |
| Matsumoto et al., 2014 | Y | Y | Y | Y | Y | Y | U | Y | Include |
| Shakir et al., 2017 | Y | Y | Y | Y | Y | Y | U | Y | Include |
| Ghysen et al., 2018 | Y | Y | Y | Y | Y | Y | U | Y | Include |
| Majumder et al., 2021 | Y | Y | Y | Y | N | NA | NA | Y | Include |
| Lardinois et al., 2022 | Y | Y | Y | Y | Y | Y | Y | Y | Include |

**Table S2. JBI Critical Appraisal of Case Series**

| **Study** | **Q1** | **Q2** | **Q3** | **Q4** | **Q5** | **Q6** | **Q7** | **Q8** | **Q9** | **Q10** | **Appraisal** |
| --- | --- | --- | --- | --- | --- | --- | --- | --- | --- | --- | --- |
| Trebesius et al., 2001 | Y | Y | Y | U | U | U | Y | U | Y | NA | Include |
| Qualia et al., 2007* | Y | Y | Y | U | U | Y | Y | U | Y | NA | Include |
| Iwańczak et al., 2006* | Y | Y | Y | U | U | Y | Y | Y | Y | Y | Include |
| Van den Bulck et al., 2005 | Y | Y | Y | U | U | U | Y | Y | Y | Y | Include |
| De Groote et al., 2005* | Y | Y | Y | U | U | Y | Y | Y | Y | Y | Include |
| Chang et al., 2024* | Y | Y | Y | U | U | Y | Y | Y | Y | Y | Include |

**Table S3. JBI Critical Appraisal of Analytical Cross-sectional Studies**

| **Study** | **Q1** | **Q2** | **Q3** | **Q4** | **Q5** | **Q6** | **Q7** | **Q8** | **Appraisal** |
| --- | --- | --- | --- | --- | --- | --- | --- | --- | --- |
| Khalilian et al., 2022 | Y | Y | Y | Y | Y | N | Y | Y | Include |
| Fritz et al., 2006 | Y | Y | Y | Y | U | N | Y | Y | Include |
| Cortez Nunes et al., 2025 | Y | Y | Y | Y | U | N | Y | Y | Include |
| Joo et al., 2007 | Y | Y | Y | Y | N | N | Y | Y | Include |
| Mohammadi et al., 2019 | Y | Y | Y | Y | N | N | Y | Y | Include |
| Chung et al., 2014 | Y | U | Y | Y | N | N | Y | Y | Include |
| Yakoob et al., 2012 | Y | Y | Y | Y | Y | N | Y | Y | Include |
| Moussa et al., 2021 | U | U | U | Y | N | N | Y | N | Include |

**Table S4. JBI Critical Appraisal of Prevalence Studies**

| **Study** | **Q1** | **Q2** | **Q3** | **Q4** | **Q5** | **Q6** | **Q7** | **Q8** | **Q9** | **Appraisal** |
| --- | --- | --- | --- | --- | --- | --- | --- | --- | --- | --- |
| Matos et al., 2022 | Y | U | Y | Y | Y | Y | Y | Y | NA | Include |
| Shafaie et al., 2020 | U | U | Y | Y | U | Y | Y | Y | NA | Include |
| Švec et al., 2000* | Y | U | Y | Y | Y | Y | Y | Y | NA | Include |
| Okiyama et al., 2005 | Y | Y | Y | Y | Y | Y | Y | Y | NA | Include |
| Boyanova et al., 2007 | Y | U | Y | Y | Y | Y | Y | Y | NA | Include |
| Liu et al., 2015* | U | U | Y | Y | Y | Y | Y | Y | U | Include |
| Iwańczak et al., 2012 | Y | U | Y | Y | U | Y | Y | Y | U | Include |

**Table S5. JBI Critical Appraisal of the Mixed Observational/Cohort Study**

| **Study** | **Q1** | **Q2** | **Q3** | **Q4** | **Q5** | **Q6** | **Q7** | **Q8** | **Q9** | **Q10** | **Q11** | **Appraisal** |
| --- | --- | --- | --- | --- | --- | --- | --- | --- | --- | --- | --- | --- |
| Taillieu et al., 2023 | Y | Y | Y | Y | U | N | Y | Y | U | Y | Y | Include |
